# *Cucumeropsis mannii* seed oil (CMSO) restores testicular mitochondrial dysfunctions by modulating the activities of dysregulated testicular mitochondrial enzymes in male albino rats exposed to bisphenol A

**DOI:** 10.1002/fsn3.4379

**Published:** 2024-08-13

**Authors:** H. A. Ogwoni, P. M. Aja, Ejike Daniel Eze, P. C. Agu, Afodun Adam Moyosore, B. A. Ale, E. U. Ekpono, J. N. Awoke, Patience N. Ogbu, O. U. Ukachi, O. U. Orji, P. C. Nweke, C. O. Egwu, E. U. Ekpono, G. O. Ewa, I. O. Igwenyi, E. U. Alum, D. C. Chukwu, Lucy Aja, G. O. Ani, C. E. Offor, O. E. Yakubu, E. Maduagwuna, J. B. Akobi, Sana Noreen, Chinaza Godswill Awuchi

**Affiliations:** ^1^ Department of Biochemistry, Faculty of Science Ebonyi State University Abakaliki Nigeria; ^2^ Department of Medical Biochemistry, Faculty of Basic Medical Sciences Cross River University of Technology (CRUTECH) Calabar Nigeria; ^3^ Department of Biochemistry, Faculty of Biomedical Sciences Kampala International University Bushenyi Uganda; ^4^ Department of Physiology, School of Medicine Kabale University Kabale Uganda; ^5^ Department of Anatomy and Cell Biology, Faculty of Health Sciences Busitema University Sukulu Uganda; ^6^ Department of Biochemistry, Faculty of Biological Sciences University of Nigeria Nsukka Nigeria; ^7^ Department of Science Laboratory Technology, Biochemistry Option Federal Polytechnic Oko Nigeria; ^8^ Department of Medical Biochemistry, Faculty of Basic Medical Sciences, College of Medicine Alex‐Ekwueme Federal University, Ndufu‐Alike Ikwo Nigeria; ^9^ Department of Science Education, Faculty of Education Ebonyi State University Abakaliki Nigeria; ^10^ Department of Biochemistry, Faculty of Sciences Federal University Wukari Nigeria; ^11^ University Institute of Diet and Nutritional Sciences University of Lahore Lahore Pakistan; ^12^ School of Natural and Applied Sciences Kampala International University Kampala Uganda

**Keywords:** bisphenol A, *Cucumeropsis mannii* seed oil, mitochondrial enzymes, mitochondrial membrane potential, nutraceutical

## Abstract

Bisphenol A, a traditional endocrine disruptor, has been implicated in male infertility. This study investigated the effect of *Cucumeropsis mannii* seed oil (CMSO) on bisphenol A (BPA)‐induced biochemical toxicity in the testicular mitochondria of male albino rats. The rats were assigned randomly to six experimental groups (*n* = 6), A, B, C, D, E, and F. Group A received 1 mL of olive oil. Groups B and C received 100 mL/kg body weight (BW) of BPA and 7.5 mL/kg BW CMSO, respectively. Rats in groups D, E, and F received preadministered doses of 100 mL/kg BW of BPA, 5 mL/kg BW of BPA, and 2.5 mL/kg BW of CMSO, respectively, followed by 6 weeks of exposure to those doses. Some mitochondrial enzymes, mitochondrial membrane potential (MMP), mitochondria testicular protein, and body weight of rats were determined using standard methods. BPA significantly reduced succinate dehydrogenase, malate dehydrogenase, isocitrate dehydrogenase, NADH dehydrogenase, and monoamine oxidase activity. Also, BPA prominently decreased the MMP, mitochondrial testicular protein, and body weight of rats. Interestingly, coadministration of BPA and CMSO restored the dysregulated activities of the enzymes and levels of other biomarkers. We postulated that CMSO may be a promising drug for treating systemic toxicity caused by environmental toxicants such as BPA.

## Graphical abstract

The study examined the effect of *Cucumeropsis mannii* seed oil (CMSO) on the biochemical toxicity produced by bisphenol A (BPA) in the testicular mitochondria of male albino rats. This study suggests that CMSO could be an effective treatment for systemic toxicity produced by environmental toxins such as BPA.

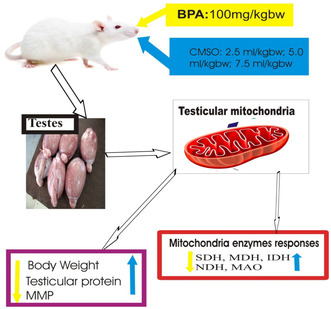

## 1 BACKGROUND

Over the years, male infertility has become a global health concern. According to Khokhar, several factors, including hormonal imbalances, genetic disorders, congenital disorders, diseases of the reproductive system, smoking, obesity, chronic alcohol abuse, long‐term marijuana use, environmental toxins, and sexually transmitted diseases, have been linked to the pathogenesis of infertility. According to Khokhar, infertile couples account for 12%−16% of all married couples worldwide. A comprehensive analysis of infertility incidence in more than 190 countries and regions globally accounted for male infertility prevalence. Bisphenol A, a classical endocrine system disruptor, has been linked to male infertility worldwide (Michelangeli et al., 2008; Ranjit et al., 2010). BPA is a polycarbonate plastic monomer used to make the linings of food cans and epoxy and polystyrene resins for plastic baby bottles (vom Saal & Hughes, 2005). According to studies, BPA is harmful to humans and mimics estrogen receptors, leading to hormonal imbalances (Santiago et al., 2021). Moreover, BPA interferes with endogenous hormones and receptors (Le et al., 2008). BPA reduces hormone secretions in the pituitary glands, including follicle‐stimulating hormone (FSH) and luteinizing hormone (LH) (Motoyama et al., 2009).

According to Ranjit et al. (2010), BPA intoxication reduces testis and prostate gland weights, serum testosterone levels, and the width and thickness of seminiferous tubules, and significantly reduces sperm count and motility. Furthermore, the mechanism of BPA migration from polycarbonate plastics and epoxy resins into food products is temperature dependent (vom Saal & Hughes, 2005). *Cucumeropsis mannii* has been reported in the literature to contain macronutrients that contribute to the health benefits of these seeds through the quality of their amino acids and fatty acids. However, knowledge of phytochemical compounds (such as phenolic compounds, which are secondary metabolites of plant metabolism) and minerals could help justify Cucumeropsis mannii seed oil (CMSO) medicinal properties (Lehucher et al., 2001). According to Osman et al. (2023), phenolic compounds have anticarcinogenic, anti‐inflammatory, antiatherosclerosis, and antitumoral properties. Reactive oxygen species (ROS) (hydroxyl and superoxide radicals, hydrogen peroxide) cause diseases such as cancer and atherosclerosis. Phytochemical substances protect the body from oxidative stress and degenerative diseases such as cancer and atherosclerosis (Dykes & Rooney, 2007). The activity of antioxidant enzymes, the organism's first line of defense against ROS, depends on minerals such as copper, zinc, manganese, and selenium (Lehucher et al., 2001) Much research has revealed that *C. mannii* seeds are a good source of essential amino acids, essential fatty acids, minerals, vitamins, polyphenols, and other important phytochemicals, which are nutraceuticals that can be used to treat various illnesses (Basharat et al., 2023; Okwundu et al., 2021). For instance, essential fatty acids such as oleic, linoleic, stearic acid, and omega‐6 fatty acids, which are the major components of *C. mannii* seeds, have antimicrobial, antioxidant, and anti‐inflammatory properties, explaining their therapeutic potential against gastrointestinal tract infections, infertility, and oxidative damage (Aja et al., 2023; Nwozo et al., 2023).

According to Agu et al. (2022), CMSO perhaps has bioactive compounds that protect the testis cell membrane and modulate tissue regeneration to attenuate the biochemistry and histological alterations against BPA‐induced testicular toxicity in rats. Aja et al. (2023) reported that *C. mannii* seed oil has considerable antioxidant potential which can be explored in therapeutic development against systemic toxicity induced by exposure to bisphenol A. Aja et al. (2023) reported that CMSO ameliorates bisphenol A‐induced adipokines dysfunctions and dyslipidemia.

In the present study, we investigated the potential protective effects of CMSO against testicular mitochondrial dysfunction brought on by dysregulated testicular mitochondrial enzymes in male albino rats.

## 2 METHODS

### 2.1 Chemicals

Bisphenol A was purchased from Riedel‐De HaenAGSeelze‐Hannover, Germany via Bristol Scientific. Bovine serum albumin (BSA), sodium trioxocarbonate (IV), sodium potassium tartrate, copper sulfate, phosphomolybdic, and phosphotungstic acid were purchased from Sigma‐Aldrich Corp. (St. Louis, MO, USA). Succinate (Sigma), nitro blue tetrazolium (Sisco Research Laboratories, SRL), ethyl acetate (Sigma), ethanol (Sigma), trichloroacetic acid (Sigma), nicotinamide adenine dinucleotide reduced (NADH) (Sigma), NADP (SRL), oxaloacetic acid (SRL), benzylamine (Sigma), and rhodamine 123 (SRL) were purchased.

### 2.2 Collection and authentication of plant material

The plant material used in this study is *C.mannii* (Abakaliki wild type: “Egusi”) seed purchased from market women in Iboko market, Izzi L.G.A., Ebonyi state. The plant was classified and authenticated by a plant taxonomist Mr. O. E. Nwankwo of the Department of Applied Biology of Ebonyi State University, Abakaliki, Nigeria.

### 2.3 Extraction of (CMSO)

The peeled *C.mannii* seeds were ground using an automated grinder. The seed oil from the ground seeds was extracted locally by mechanical pressing using a mortar and pestle. Drops of water were added to enhance the release of oil, as water helped to rupture the cells. Water binds to hydrocolloids (gum and mucilage) (Dror et al., 2006) and is allowed to sediment. The extract was left to stand undisturbed for 2–3 days to sediment and was separated by the decantation method to obtain a purer form of the oil, which was then stored in clean bottles for use (Hamed & Bahareh, 2012: Agu et al., 2022).

### 2.4 Preparation of BPA stock solution

The BPA pellets were made into powder using mortar and pestle. The solution of BPA was made by dissolving 5 g of BPA in 100 mL of olive oil according to Alboghobeish et al. (2019) and Aja et al. (2023). Olive oil served as the vehicle and was administered to the normal control.

### 2.5 Acute toxicity of CMSO

The acute toxicity of CMSO in male Wistar albino rats was determined by OECD guideline no. 425 (OECD, 2008). The acute toxicity study was conducted using the limit dose test of the up‐and‐down procedure according to OECD/OCDE guidelines no. 425. Male albino Wistar rats (aged 2 months old) were used for the study and they were acclimatized for 7 days to the laboratory condition before the commencement of the experiment. Following an overnight fast of a female rat, 50 mL/kg of the CMSO was administered to it orally. The animal was thereafter strictly observed for physical or behavioral changes for the first 30 min, following extract administration, and then periodically (with special attention given during the first 4 h) for the next 24 h and then daily thereafter, for 14 days. Food was given after 3–4 h of CMSO administration. Following the survival of the first rat, other four male rats were recruited and fasted for 4 h. They were subsequently administered the same dose of CMSO followed by similar strict observation which was continued for further 14 days for any signs of toxicity (Eleazu et al., 2021; Saleem, 2017; Tadesse, 2014). At the limit test dose of 50 mL/kg, the rats did not show any indication of gross physical or behavioral changes such as hair erection, reduction in feeding, and motor activities within the 24‐h monitoring period as well as within the 14 days. For this reason, 10% of the limit dose (5 mL/kg) was selected as the middle/intermediate dose, half of it (2.5 mL/kg) was selected as the lower dose, and 1.5 times the middle dose (7.5 mL/kg) was taken as the higher dose based on OECD guideline no. 425 (OECD, 2008).

### 2.6 Experimental animals

The experimental animals used in this study were albino Wistar rats purchased from the Animal House of the Faculty of Veterinary Medicine, University of Nigeria, Nsukka, Enugu, Nigeria. The rats were handled as recommended by the International Standard Procedure for Experimental Animal Handling of the National Institute of Health (NIH), USA (NIH publications no. 80‐23, revised in 1996) and adopted by Aja et al. (2023) which has been adopted by the Biochemistry Department, Ebonyi State University, Nigeria was followed after due approval by the Departmental Ethical Committee (ethical approval number EBSU/BCH/ET/19/001).

### 2.7 Experimental design

A total of 48 male albino Wistar rats were used for this study. The rats were randomly assigned into six experimental groups of A, B, C, D, E, and F with eight rats in each group. Groups A, B, and C are the control groups while groups D, E, and F are the treatment groups.

**Table:** 

Group A:	Rats received 1 mL of olive oil only and served as the normal control known to be control group 1 (CG1)
Group B:	Rats received 100 mg/kg body weight of BPA orally and served as the BPA control group known to be control group 2 (CG2) (Samova et al., 2018)
Group C:	Rats received 7.5 mL/kg body weight of *Cucumeropsis mannii* seed oil (CMSO) orally and served as the CMSO control group known to be control group 3 (CG3)
Group D:	Rats were preadministered 100 mg/kg body weight of BPA and were treated with 7.5 mL/kg body weight of CMSO and served as treatment group 1 (TG1)
Group E:	Rats were preadministered 100 mg/kg body weight of BPA and were treated with 5 mL/kg body weight of CMSO and served as treatment group 2 (TG2)
Group F:	Rats were preadministered 100 mg/kg body weight of BPA and were treated with 2.5 mL/kg body weight of CMSO and served as treatment group 3 (TG3)

Administration of both BPA and CMSO was concurrently by oral intubation once every day for 6 weeks.

### 2.8 Tissue sample collection

The animals were sacrificed by cervical dislocation under mild anesthesia. Scrotal sacs were dissected using a sharp blade to remove the right and left testicles and their morphology (length, width, and volume) was examined in each group. The testicular volume was calculated using the following equation: volume = (*D*2/4 × π) *L* × *K* (length [*L*], width [*D*], *K* = 0.9).

### 2.9 Isolation of testicular mitochondria

Testes were minced in freshly isolated medium (70 mM sucrose, 220 mM mannitol), 10 mM 2‐[4‐(2 hydroxyethyl) piperazine‐1‐yl] ethane sulfonic acid (HEPES), 1 mM ethylenediaminetetraacetic acid (EDTA) buffer (pH 7.4, 2.5 mM MgCl2 [SRL]), 0.5 mM. The homogenate was centrifuged for 10 min at 500 × *g*. The supernatant was retained, and a fresh isolation medium was used to wash the pellet and then recover using the initial supernatant (Aja et al., 2023; Bello et al., 2021; Park & Pang, 2021). Pooled fractions were centrifuged for 10 min at 500 × *g* and supplemented with protease and phosphatase inhibitors. The obtained supernatant was centrifuged for 15 min at 5000 × *g* to obtain a mitochondrial pellet. Pure mitochondria were recovered by centrifugation at 4°C for 10 min at 12,000 × *g*. The supernatant containing the mitochondria was collected and used for biochemical analyses (Aja et al., 2023; Bello et al., 2021; Park & Pang, 2021).

Testes were minced in a fresh isolation medium (70 mM sucrose, 220 mM mannitol), 10 mM 2‐[4‐(2 hydroxyethyl) piperazin‐1‐yl] ethanesulfonic acid (HEPES), 1 mM EDTA buffer at pH 7.4, 2.5 mM MgCl_2_ (SRL), 0.5 mM KH_2_PO_4_ and gently homogenized manually using a Dounce glass homogenizer with a loose‐fitting pestle. The homogenate was centrifuged at 500 × *g* for 10 min. The supernatant fraction was retained, whereas the pellet was washed with a fresh isolation medium and recovered by the initial supernatant fraction and pooled fractions were centrifuged at 500 × *g* for 10 min and supplemented with protease and phosphatase inhibitors. The supernatant thus obtained was centrifuged at 5000 × *g* for 15 min to obtain the mitochondria pellet. The purified mitochondria were obtained by centrifugation at 12,000 × *g* for 10 min at (4°C). The supernatant containing mitochondria was collected and used for various biochemical analyses (Sayeed et al., 2006).

### 2.10 Measurement of testicular mitochondrial enzymes

*Succinate dehydrogenase* (SDH) was measured by the method of Anjum et al. (2011). The assay mixture consisted of sodium succinate (Sigma) 0.01 M and a 3‐mL mitochondrial fraction. The samples were kept at 37°C for 15 min and then 0.1 M nitro blue tetrazolium (NBT) as well as 2.5 mg/mL buffer (Sisco Research Laboratories (SRL)) was added. After 10 min of incubation, 1 mL of solution A (ethyl acetate: Ethanol: Trichloroacetic acid (TCA) (SRL); 5:5:1; v/v/w) was added to the reaction mixture and centrifuged for 1 min at 22,000 × *g*. The absorbance was recorded at 490 nm against blank. The activity was calculated as nmol formazan formed/min/mg protein.

*Malate dehydrogenase activity* was measured by the method of Anjum et al. (2011). The reaction mixture consisted of 0.25 M sucrose buffer at pH 7.4, 3.75 mM nicotinamide adenine dinucleotide reduced (NADH) (Sigma), 6 mM oxaloacetic acid (SRL), and 3‐mL mitochondrial fraction added. The change in absorbance was recorded at 340 nm for 3 min. Enzyme activity was calculated as nmol NADH oxidized/min/mg protein using the molar extinction coefficient of 6.22 × 10^3^ M^−1^ cm^−1^.

*Isocitrate dehydrogenase* (IDH) was measured by the method of Anjum et al. (2011). The reaction mixture consisted of 0.2 M Tris buffer at pH 8.5 (SRL), 4.6 mM isocitrate solution (Sigma), 3‐mL mitochondrial fraction, and 2.88 mM nicotinamide adenine dinucleotide phosphate (NADP) (Sigma). The change in absorbance was recorded at 340 nm for 3 min. The enzyme activity was calculated as nmol NADPH oxidized/min/mg protein using a molar extinction coefficient of 6.22 × 10^3^ M^−1^ cm^−1^.

*NADH dehydrogenase* (NDH) was measured by the method of El‐Beshbishy et al. (2013). The reaction mixture consisted of 0.05 M sodium phosphate buffer at pH 7.6, 0.25 M sucrose, 3‐mL mitochondrial fraction, and 0.28 mM NADH. The change was recorded at 340 nm for 3 min. The enzyme activity was calculated as nmol NADH oxidized/min/mg protein using a molar extinction coefficient of 6.22 × 10^3^ M^−1^ cm^−1^.

*Monoamine oxidase* (MAO) was measured by the method of Zhang et al. (2003). The reaction mixture consisted of 0.25 M sucrose buffer at pH 7.4, 8 mM benzylamine (Sigma), and mitochondrial fraction. The reaction mixture was incubated for 40 min at 37°C. Then, 1‐mL perchloric acid (SD Fine Chem Ltd., Mumbai, India) was added and the reaction product was extracted with 3‐mL cyclohexane (Hi‐Media) by centrifugation at 600 g for 10 min, and the absorbance of the supernatant was measured at 242 nm. The enzyme activity was expressed as nmol benzaldehyde formed/min/mg protein.

### 2.11 Measurement of testicular mitochondrial membrane potential

Mitochondrial membrane potential was determined using the mitochondrial uptake of the cationic fluorescent dye Rh 123. For incubation of the mitochondrial suspensions (0.5 mg protein/ml), the tubes were mildly shaken at 37°C with 1.5 μM Rh 123 for 10 min. Following that, the Elmer LS‐50B luminescence fluorescence spectrophotometer was applied at emission and excitation wavelengths of 490 and 535 nm, respectively, to estimate the fluorescence (Baracca et al., 2003).

### 2.12 Estimation of total testicular proteins

The total testicular proteins were determined using the method of Adedara et al. (2013) and using BSA, a globular protein (1 mg/mL) as the standard. The process started by adding 4.5 mL of Lowry working reagent, which is 2% Na_2_CO_3_ in 0.1 N NaOH, 1% sodium potassium tartrate in H_2_O, 0.5% CuSO_4_ .5 H_2_O in water and incubating for 10 min at room temperature. 0.2 mL of Folin phenol reagent, which is 0.2 N phosphomolybdic and 0.2 N phosphotungstic acids, was added and left for 30 min and absorbance was read at 750 nm in the spectrophotometer.

### 2.13 Statistical analysis

The statistical analyses were performed using Graph Pad Prism 5.04 (GraphPad, La Jolla, CA, USA). Data were expressed, as mean ± SD. One‐way ANOVA with Tukey's test was used for the statistical tests. In general, *p* < .05 was considered the statistical level of significance.

## 3 RESULTS

### 3.1 Investigations on testicular mitochondrial marker enzymes

**FIGURE 1:**
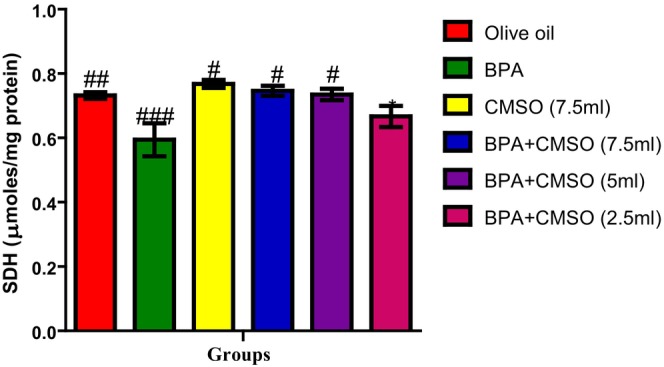
Effect of CMSO on succinate dehydrogenase activity of testicular mitochondria in BPA‐induced testicular toxicity in albino rats. Data are shown as mean ± SD (*n* = 6). Mean values with the different signs are significantly different at *p* < .05 (*, #, ##, ###). BPA, bisphenol A; CMSO, *Cucumeropsis mannii* seed oil.

As shown in Figure 1, BPA administration significantly decreased the activity of succinate dehydrogenase in the testicular mitochondria when compared with the normal control group. However, coadministration of CMSO significantly reversed this abnormal decrease in the activity of SDH when compared with the BPA group.

**FIGURE 2:**
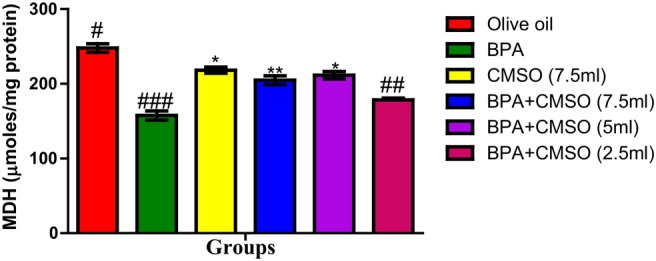
Effect of CMSO on malate dehydrogenase activity in testicular mitochondria of BPA‐induced testicular toxicity in albino rats. Data are shown as mean ± SD (*n* = 6). Mean values with the different signs are significantly different at *p* < .05 (*, #, ##, ###). BPA, bisphenol A; CMSO, *Cucumeropsis mannii* seed oil.

As revealed in Figure 2, a significant decrease in malate dehydrogenase activity was also observed in the testicular mitochondria in rats induced with BPA over the normal control group. Interestingly, this aberration in the activity of this enzyme was normalized with the coadministration of CMSO with the BPA.

**FIGURE 3:**
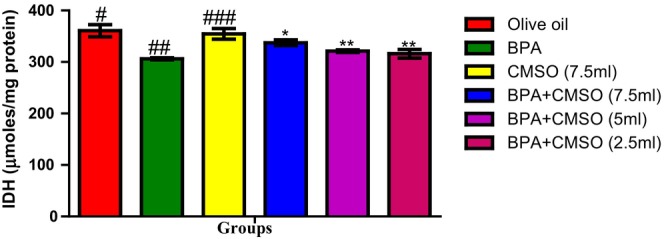
Effect of CMSO on isocitrate dehydrogenate activity of testicular mitochondria in BPA‐induced testicular toxicity in albino rats. Data are shown as mean ± SD (*n* = 6). Mean values with the different signs are significantly different at *p* < .05 (*, #, ##, ###). BPA, bisphenol A; CMSO, *Cucumeropsis mannii* seed oil.

Figure 3 shows that BPA administration resulted in a significant decrease in isocitrate dehydrogenate activity when compared with the activity of the same in the normal control rats. The CMSO‐treated group showed a significant increase in isocitrate dehydrogenase activity when compared with that of BPA‐treated group.

**FIGURE 4:**
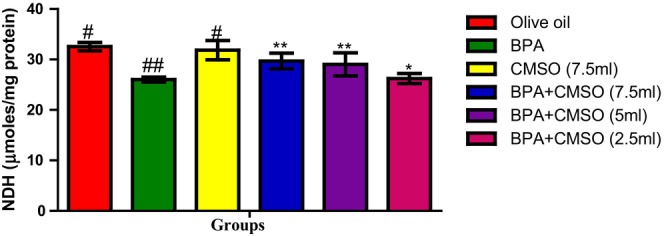
Effect of CMSO on NADH dehydrogenase activity of testicular mitochondria in BPA‐induced testicular toxicity in albino rats. Data are shown as mean ± SD (*n* = 6). Mean values with the different signs are significantly different at *p* < .05 (*, #, ##, ###). BPA, bisphenol A; CMSO, *Cucumeropsis mannii* seed oil.

Similarly, the result in Figure 4 shows that induction with BPA resulted in a significant decrease in NADH dehydrogenase activity when compared with the normal control group. The CMSO group (BPA + CMSO) showed a significant increase in this enzyme activity when compared with that of the BPA‐treated group.

**FIGURE 5:**
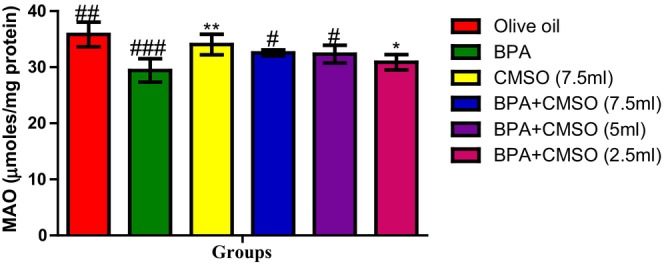
Effect of CMSO on monoamine oxidase activity in testicular mitochondria in BPA‐induced testicular toxicity in albino rats. Data are shown as mean ± SD (*n* = 6). Mean values with the different signs are significantly different at *p* < .05 (*, #, ##, ###). BPA, bisphenol A; CMSO, *Cucumeropsis mannii* seed oil.

In Figure 5, a significant decrease in MAO activity was also observed in BPA‐treated rats over the normal control group. Conversely, rats of the group treated with BPA + CMSO showed significant restoration of MAO activity when compared with that of the BPA‐treated group.

### 3.2 Investigations of testicular mitochondrial membrane potential

**FIGURE 6:**
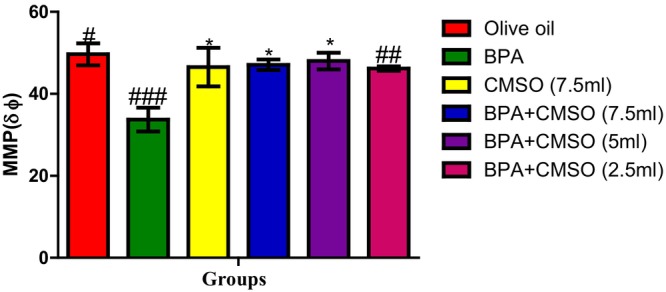
Effect of CMSO on mitochondrial membrane potential level of testicular mitochondria in BPA‐induced testicular toxicity in albino rats. Data are shown as mean ± SD (*n* = 6). Mean values with the different signs are significantly different at *p* < .05 (*, #, ##, ###). BPA, bisphenol A; CMSO, *Cucumeropsis mannii* seed oil.

Figure 6 reveals a significant decrease in mitochondrial membrane potential levels in BPA‐treated rats over the normal control group. Interestingly, mitochondrial membrane potential level was significantly elevated in rats of the group treated with BPA + CMSO, when compared with that of BPA‐treated group.

### 3.3 Investigations on total testicular proteins and body weights

**FIGURE 7:**
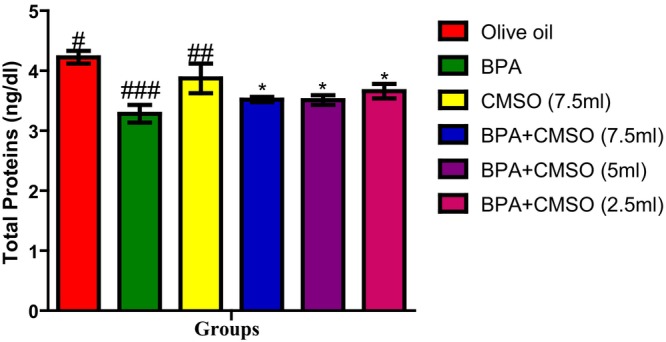
Effect of CMSO on total testicular protein in BPA‐induced testicular toxicity in albino rats. Data are shown as mean ± SD (*n* = 6). Mean values with the different signs are significantly different at *p* < .05 (*, #, ##, ###). BPA, bisphenol A; CMSO, *Cucumeropsis mannii* seed oil.

BPA administration as shown in Figure 7 resulted in a significant decrease in testicular protein when compared with the normal control group. This abnormal decrease in the testicular protein of the mitochondria was, however, significantly reversed with coadministration of CMSO (BPA + CMSO) when compared with that of the BPA‐treated group.

**FIGURE 8:**
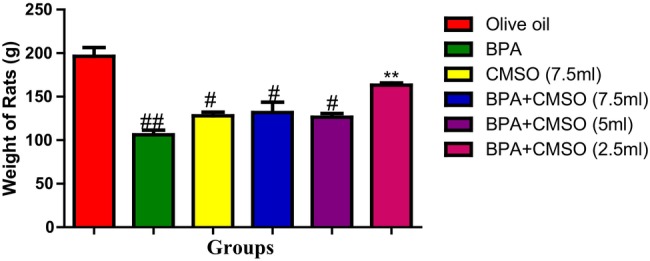
Effect of CMSO on body weight of the rats induced with BPA. Data are shown as mean ± SD (*n* = 6). Mean values with the different signs are significantly different at *p* < .05 (*, #, ##, ###). BPA, bisphenol A; CMSO, *Cucumeropsis mannii* seed oil.

Additionally, Figure 8 reveals that BPA treatment resulted in a significant reduction in the body weight of the rats when compared with the normal control group. Coadministration of CMSO at the highest dosage (BPA + CMSO) showed a significant increase in the body weight of rats when compared with that of the BPA‐treated group.

## 4 DISCUSSION

In this present study, it was observed that BPA significantly decreased the activities of testicular mitochondrial enzymes such as succinate dehydrogenase, malate dehydrogenase, isocitrate dehydrogenase, monoamine oxidase, and NADH dehydrogenase. These present findings agreed with the findings of Anjum et al. (2011) who reported that melatonin ameliorates bisphenol‐A‐induced biochemical toxicity in testicular mitochondria of mice and showed a decrease in the activities of mitochondrial enzymes such as succinate dehydrogenase, malate dehydrogenase, isocitrate dehydrogenase, monoamine oxidase, and NADH dehydrogenase.

However, Chattopadhyay et al. (2010) also reported the toxicity and perturbation of mitochondrial membrane induced by BPA that ultimately decreased mitochondrial enzymes and altered reproductive and endocrine function, thus impairing steroidogenesis and spermatogenesis. Furthermore, this present study agreed with the findings of Bindhumol et al. (2003) and Rashid et al. (2009) who reported that BPA induced oxidative stress in different tissues in rats that ultimately leads to the generation of ROS such as hydroxyl radical, peroxynitrite anion, nitric oxide, singlet oxygen, and the peroxyl radical. Similarly, Anisimov and Wang (2006) and Huang et al. (2002) reported the ameliorative potentials of naringin against bisphenol‐A‐induced biochemical toxicity in testicular mitochondria of the mouse. The observed decrease in the activity of mitochondrial marker enzymes in this study could be attributed to slower enzyme production, increased metabolite buildup, and toxicant binding to the active site of enzymes. Their disruption in testicular mitochondria as a result of BPA exposure shows an imbalance in mitochondrial energetics, particularly ATP generation. Reduced ATP generation can be a primary event induced by a direct influence on mitochondria or a secondary event caused by changed metabolism as a result of any of the compound's interactions inside the cell (Nakagawa & Tayama, 2000).

The decrease in mitochondrial marker enzyme activity might be attributed to oxidative damage caused by the respiratory chain complexes I and III, which produce superoxide anions, which impact ATP generation. As a result, ATP is generated through oxidative phosphorylation in mitochondria, which is mediated by membrane‐bound protein complexes such as NADH‐dehydrogenase (complex I), succinate dehydrogenase (complex II), cytochrome c oxidoreductase (complex III), and total ATPases. Succinate dehydrogenase solely provides electrons to the electron transport chain (ETC), whereas NADH‐dehydrogenase is involved in both proton translocation and electron transfer. A defect in any of the oxidative metabolism enzyme complexes can cause mitochondrial cytopathy and the opening of the mitochondrial permeability transition pore, which allows the membrane potential to dissipate, causing uncoupling of oxidative phosphorylation and thus impair cellular ATP production.

The activity of mitochondrial enzymes such as succinate dehydrogenase, malate dehydrogenase, isocitrate dehydrogenase, NADH dehydrogenase, and monoamine oxidase was restored and greatly boosted when CMSO was coadministered. The chemical constituents of CMSO, such as omega‐6 fatty acids, omega‐3 fatty acids, palmitic acids, stearic acids, oleic acids, linoleic acids, and other monounsaturated and polyunsaturated fatty acids, have been reported to protect the functional and structural integrity of cell membranes as well as the maintenance of membrane phospholipids, which may explain the restoration of mitochondrial enzymes.

Furthermore, BPA reduced the potential of mitochondrial membranes substantially. These findings matched those of Huc et al. (2012), who found that BPA caused a mitochondrial malfunction in isolated rat hepatocytes, including a decrease in mitochondrial transmembrane potential and altered cellular oxidation–reduction. Similarly, Liu et al. (2014) found that BPA produced mitochondria‐mediated apoptosis in hepatic cells, as well as disturbance of liver tissues and enzymes (AST, ALP, ALT) and ETC impairment. However, the current studies revealed that BPA causes toxicity and inhibits oxidative phosphorylation and the generation of ROS (Xia et al., 2005).

As a result, the decrease in MMP may be due to the accumulation of hydroxyl radicals via the development of pro‐oxidative status, affecting the difference in electric potential between the interior and exterior of a biological cell (Callies et al., 2011), reducing mitochondrial membrane motility, signaling mechanisms, and inhibiting mitochondrial ion transport, which may eventually impair the functionality of membrane channels such as sodium ions (Na^+^) and potassium ions (K^+^). As a result, the coadministration of CMSO resulted in a considerable rise in MMP levels. CMSO's therapeutic effectiveness and antioxidant capability may have contributed to this rise by repairing the structure and function of mitochondrial membrane phospholipids.

Because the membrane potential initially permits a cell to act as a battery, giving electricity to run a range of molecular devices implanted in the membrane, MMP is critical for mitochondrial mobility. Second, the membrane potential is employed to convey messages between various regions of a cell in electrically excitable cells like neurons and muscle cells (Marieb & Hoehn, 2014) Thus, signals are formed by opening or shutting ion channels at a single spot in the membrane, resulting in a local change in the membrane potential, which can be immediately influenced by either nearby or more distant ion channels in the membrane (Marieb & Hoehn, 2014). As a result of the potential shift, the ion channels might open or close, replicating the signal and resulting in physiological actions.

BPA, in particular, reduced the quantity of testicular protein. These findings are comparable to those of Anjum et al. (2011), who found that bisphenol‐A‐induced biochemical damage in mouse testicular mitochondria resulted in a negative reduction in testicular protein levels. Furthermore, the findings of this investigation were comparable with those of prior studies, in which BPA produced a significant drop in testicular protein levels as well as a decrease in the hypothalamic–pituitary–gonadal axis, resulting in impaired Sertoli cell activity (Geens et al., 2012). Induction of BPA produced oxidative stress and alteration of the steroidogenic activity of Leydig cells, according to Alonso‐Magdalena et al. (2008), which resulted in reduced spermatogenesis. However, the reduction in testicular total protein in BPA‐treated rats could be due to BPA's antagonistic effects on reproductive organ proteins and lipids, resulting in stimulatory and inhibitory factors present in the testicular fluid that selectively change protein production, lowering testicular synthetic activity and thus affecting spermatogenesis (Thomas & Dong, 2006).

The efficacy of CMSO, which has been shown to contain antioxidants and minerals such as iron, zinc, copper, and selenium that serve as coenzymes to enhance antioxidant capacity and restore the synthetic function of the protein, may explain why CMSO cotreatment significantly increased the level of testicular protein (Thomas & Dong, 2006). A drop‐in hormone biosynthesis, such as FSH and LH, a decrease in enzyme synthesis, a decrease in hemoglobin and myoglobin production, and a change in fluid balance management, such as albumin and globulins, are all metabolic consequences (Thomas & Dong, 2006).

Furthermore, rats' body weight was dramatically reduced by BPA. These findings matched those of Gurmeet et al. (2014), who found that following BPA exposure, rats' body weight reduced somewhat when compared to the control group. Miao et al. (2015) found a substantial (*p* > .01) reduction in body weight and testicular volume in rats when compared to the control group. As a result, several prior researchers found that when male rats were given BPA, their body weight did not alter significantly at a modest dose of the extract (Korkmaz et al., 2010; Nanjappa et al., 2012; Norazit et al., 2012). As a result, their findings did not match those of the current study, which found a substantial increase at a modest dosage of CMSO (2.5 mL). This discrepancy might be caused by the extract used.

However, the significant reduction in body weight observed in this study could be due to a reduction in testicular weight, which could be attributed to a reduction in the size of seminiferous tubules and spermatogenic cells, or it could be due to reduced bioavailability of gender hormones, which could indicate a male reproductive endocrine stipulation. The mass of spermatogenic cells is proportional to the weight and volume of the testis. As a result, decreasing testis weight impairs spermatogenesis activity, resulting in fewer germ cells. Furthermore, BPA poisoning reduced the body weight of the experimental rats by causing damage to key components in the testis, such as proteins. CMSO cotreatment, on the other hand, considerably boosted the body weight of rats, possibly due to CMSO's therapeutic efficacy and protective potential. As a consequence, the findings of this investigation were consistent with those of Anjum et al. (2011), who found that BPA exposure resulted in a substantial decrease in testicular weight, volume, and indices, but that coadministration of melatonin significantly enhanced testicular weight and volume.

## 5 CONCLUSIONS

In this study, we reported that BPA caused mitochondrial toxicity in the testicular tissue. It had an impact on the activities of antioxidant enzymes as well as essential mitochondrial bioenergetic enzymes. Rats exposed to BPA also experienced changes in their body weight, total testicular protein, and mitochondrial membrane potential, suggesting how susceptible the mitochondrial membrane is to exposure. Interestingly, *Cucumeropsis mannii* seed oil reversed mitochondrial damage caused by BPA. In addition to restoring the decreased levels of testicular protein, body weight, and mitochondrial membrane potential in rats, CMSO protected the mitochondria's essential bioenergetic enzymes from damage. Thus, *Cucumeropsis mannii* seed oil may be a potent protector of mitochondrial dysfunction due to exposure to toxicants. This leguminous crop and its oil can be subjected to clinical trials against male infertility and other BPA‐induced toxicities and other environmental toxicants with similar structures. Additional studies such as the transgenerational protective effect of the oil on the male testicular health of the first filial (F1) generation from BPA‐exposed mothers should be done. Also, the specific bioactive components of the oil responsible for its specific biological activity should be explored.

## AUTHOR CONTRIBUTIONS

**H. A. Ogwoni:** Project administration (equal); visualization (equal); writing – original draft (equal). **P. M. Aja:** Conceptualization (lead); supervision (lead); writing – review and editing (lead). **Ejike Daniel Eze:** Funding acquisition (equal); software (equal). **P. C. Agu:** Data curation (equal); validation (equal). **Afodun Adam Moyosore:** Formal analysis (equal); validation (equal). **B. A. Ale:** Investigation (equal); writing – original draft (equal). **Ekpono Ezebuilo Ugbala:** Methodology (equal); resources (equal); validation (equal). **J. N. Awoke:** Methodology (equal); resources (equal). **Patience N. Ogbu:** Data curation (equal); methodology (equal). **Felix Emmanuel Nwite:** Investigation (equal); project administration (equal). **O. U. Ukachi:** Formal analysis (equal); investigation (equal). **O. U. Orji:** Writing – review and editing; methodology (equal). **P. C. Nweke:** Software (equal); validation (equal); visualization (equal). **C. O. Egwu:** Conceptualization (equal); methodology (equal). **E. U. Ekpono:** Investigation (equal); writing – original draft (equal). **G. O. Ewa:** Investigation (equal); visualization (equal). **I. O. Igwenyi:** Validation (equal); data curation (equal); visualization (equal). **C. E. Offor:** Supervision (equal). **Lucy Aja:** Project administration (equal); validation (equal). **Onyedika Gabriel Ani:** Conceptualization (equal); methodology (equal). **Ekenechukwu K. Maduagwuna:** Investigation (equal); project administration (equal). **O. E. Yakubu:** Funding acquisition (equal); formal analysis (equal). **J. B. Akobi:** Funding acquisition (equal); resources (equal). **Sana Noreen:** Funding acquisition (equal); software (equal). **Chinaza Godswill Awuchi:** Visualization (equal); writing – original draft (equal).

## FUNDING INFORMATION

This study did not receive any form of external funding.

## CONFLICT OF INTEREST STATEMENT

The authors declare that there is no conflict of interest.

## ETHICS STATEMENT

This study received ethical approval number: EBSU/BCH/ET/19/001 from the ethical committee of the Department of Biochemistry, Ebonyi State University, Nigeria.

## CONSENT FOR PUBLICATIONS

All the authors agreed to publish the current version of the research.

## Data Availability

The data and materials are available on request.
